# The SNARE Protein CfVam7 Is Required for Growth, Endoplasmic Reticulum Stress Response, and Pathogenicity of *Colletotrichum fructicola*

**DOI:** 10.3389/fmicb.2021.736066

**Published:** 2021-10-14

**Authors:** Sizheng Li, Shengpei Zhang, Bing Li, He Li

**Affiliations:** ^1^Key Laboratory of National Forestry and Grassland Administration for Control of Diseases and Pests of South Plantation, Hunan Provincial Key Laboratory for Control of Forest Diseases and Pests, Key Laboratory for Non-Wood Forest Cultivation and Conservation of Ministry of Education, Central South University of Forestry and Technology, Changsha, China; ^2^School of Agricultural Sciences, Zhengzhou University, Zhengzhou, China

**Keywords:** SNARE protein, homotypic vacuole fusion, stress response, pathogenicity, *C. fructicola*

## Abstract

The tea-oil tree *Camellia oleifera* is native to China and is cultivated in many parts of southern China. This plant has been grown for over 2,000 years, mainly for its high-quality cooking oil. Anthracnose is the main disease of tea-oil tree and results in a huge loss annually. *Colletotrichum fructicola* is a major pathogen causing anthracnose on tea-oil tree. In a previous study, we characterized that the bZIP transcription factor CfHac1 controlled the development and pathogenicity of *C. fructicola*. Here, we identified and characterized the function of *CfVAM7* gene, which was significantly downregulated at the transcriptional level in the Δ*Cfhac1* strain under dithiothreitol stress. Targeted gene deletion revealed that CfVam7 is important in growth, pathogenicity, and responses to endoplasmic reticulum-related stresses. Further analysis revealed that CfVam7 is required for appressorium formation and homotypic vacuole fusion, which are important for fungal pathogen invasion. Cytological examinations revealed that CfVam7 is localized to vacuole membranes in the hyphal stage. The Phox homology (PX) and SNARE domains of CfVam7 were indispensable for normal cellular localization and biological function. Taken together, our results suggested that CfVam7-mediated vacuole membrane fusion promotes growth, stress response, and pathogenicity of *C. fructicola*.

## Introduction

*Camellia oleifera* is a ligneous plant native to China, mainly used to produce edible oil materials (Li et al., 2016). Previous studies have demonstrated that *Colletotrichum fructicola* is the prominent pathogen causing *Ca. oleifera* anthracnose. We recently demonstrated that CfSnf1, CfSte50, and CfGcn5 were involved in pathogenicity by regulating the appressorium formation in *C. fructicola* (Zhang et al., 2019, 2021; Chen et al., 2020). Transcription factor of CfSte12 and the MAP Kinase CfPmk1 are key regulators of pathogenesis of *C. fructicola* (Liang et al., 2019; Liu et al., 2021). Shang et al. (2020) identified a novel effector CfEC92 of *C. fructicola*, which contributes to glomerella leaf spot virulence by suppressing plant defenses at the early infection phase. In *Colletotrichum higginsianum*, ChSat4 was important for intracellular K^+^ accumulation and infection morphogenesis in *C. higginsianum* (Yang et al., 2018). Despite this knowledge, the underlying pathogenic mechanisms of *C. fructicola* remain largely unclear, which severely hinders the prevention and management of *Ca. oleifera* anthracnose (Weir et al., 2012; Li et al., 2014; Li et al., 2017; Li et al., 2019c).

Membrane fusion has an important regulatory effect on the vital functions of organisms (Su et al., 2019). The macromolecular and granular substances in cells cannot freely cross the cell membrane, and vesicles are required for their transmembrane transport (Chen et al., 2015). The soluble *N*-ethylmaleimide-sensitive fusion protein attachment protein receptor (SNARE) plays an important role in the vesicular trafficking and fusion in eukaryotes. The vesicular membrane SNAREs (v-SNAREs) and the target membrane SNAREs (t-SNAREs) could form a SNARE complex to bring the membranes into close proximity, consequently inducing membrane fusion (Zhang and Wang, 2004). All SNAREs share a common domain consisting of about 60 amino acids, which determines the characteristics of SNAREs and mediates the formation of the core complex. SNAREs also have flanking sequences that link SNAREs to the cell membrane and mediate protein–protein interactions (Jahn and Südhof, 1999). Vam7, which belongs to the t-SNARE protein family, plays a very important role in regulating the growth, development, and pathogenicity of plant pathogens (Dou et al., 2011; Zhang et al., 2016; Li et al., 2019a).

Our team has previously investigated CfHac1, a transcription factor of bZIP, in *C. fructicola* and found that CfHac1 participates in regulating the growth, sporulation, appressorium formation, pathogenicity, and endoplasmic reticulum (ER) stress response process (Yao et al., 2019). We further analyzed the transcriptome of *CfHAC1* gene knockout mutant strain of *C. fructicola* under ER stress induced by dithiothreitol (DTT). The results showed no expression of eight genes, including A01015, A00654, A13874, A05547, A12256, A12707, A09452, and A11731, of which A11731 was the coding gene encoding Vam7 protein, a subunit of the SNARE complex (Li and Li, 2020). This study aimed to investigate the biological function of the A11731 gene *CfVAM7* via various techniques including gene knockout, further providing potential target sites for the development of new fungicides. Here, we characterized the roles of CfVam7 in forest pathogen of *C. fructicola* for the first time, which was regulated by transcription factor CfHac1. And we also provided evidence that the Phox homology (PX) and SNARE domains of CfVam7 were indispensable for normal cellular localization and biological function.

## Materials and Methods

### Strains and Culture Conditions

Wild-type *C. fructicola* was separated and stored by our laboratory, and the whole-genome sequencing has already been completed (Liang et al., 2020). Plasmids of yeast strains XK-125 and pYF11 were provided by Nanjing Agricultural University. All of the strains were cultured on potato dextrose agar (PDA) plate at 28°C in darkness, unless the medium is mentioned.

### qRT-PCR and Identification of Target Gene

RNA was extracted from the wild-type *C. fructicola* and the mutant strain (Δ*Cfhac1*), and the first strand of cDNA was synthesized by the TIANscript RT Kit (Tiangen Biotechnology Co., Ltd., Beijing, China). The expression of the target gene was normalized to *ACTIN*, and then the relative expression ratio of the target gene in the mutant strain to wild-type strain was estimated by acquiring the Ct value after amplification by QuantStudio 3 (Thermo Fisher Scientific, Waltham, MA, United States). Each sample was prepared in triplicate, and the experiments were repeated three times. As the protein encoded by A11731 gene was an orthologous protein of the ScVam7 protein of *Saccharomyces cerevisiae*, it was named CfVam7.

### Sequence or Bioinformatics Analysis

The Vam7 proteins in of other fungi were obtained from the National Center for Biotechnology Information (NCBI) database.^1^ The neighbor-joining (N-J) phylogenetic tree was constructed by the N-J method using the amino acid sequences of CfVam7 and orthologous protein of other fungi, and the phylogenetic relationship was explored. The domains of CfVam7 were predicted by SMART.^2^

### Gene Deletion, Complementation, and Domain Deletion Assays

The overlap method was used to contrast *CfVAM7* gene knockout fragment, as our previous description (Li et al., 2021). The genomic DNA of wild-type CFLH16 strain was used as the template of DNA. Primers including CfVam7-1F/CfVam7-2R and CfVam7-3F/CfVam7-4R were used for the amplification to acquire the DNA fragments of about 1 kb at upstream and downstream of the coding region of *CfVAM7* gene, respectively. The primers Hyg-F/Hyg-R were used for the amplification of the hygromycin resistance gene (*HPH*) fragment. Finally, the upstream and downstream fragments of *CfVAM7* gene and *HPH* gene fragment were used as templates, and the primers CfVam7-1F/CfVam7-4R were used again for further PCR amplification to acquire *CfVAM7* gene knockout fragment. The PEG-mediated protoplast transformation method was used to acquire the gene knockout mutant. The preparation of protoplast and genetic transformation was performed according to the method reported by Li et al. (2021). The *CfVAM7* knockout fragment was transformed into the protoplast of wild-type CFLH16, and the mutants were screened and cultured on a hygromycin-containing TB3 culture medium. For subsequent verification by agarose gel electrophoresis, the inner primers of *CfVAM7* gene, CfVam7-7F/CfVam7-8R, and the outer primers of *CfVAM7* gene, CfVam7-5F/H855R, were used to amplify the transformant DNA. When the strips with correct sizes could not be amplified by the PCR using inner primers but could be amplified by PCR using outer primers, the mutant strain was identified. The sequences of primers are shown in Supplementary Table 1.

For complementation strain, the CfVam7-9F and CfVam7-10R primers were designed, and the complementary fragments that included *CfVAM7* gene and promoter sequences were amplified by PCR. The PCR products were purified and then co-transformed into the yeast competent cell XK-125 with pYF11 vector that was linearized using *Xho*I [containing bleomycin (BLE)-resistant gene and green fluorescence protein (GPF) gene] to form the complementary carrier pYF11:*CfVAM7* (Zhang et al., 2021). The yeast cells were cultured on SD-Trp medium for screening, and the primers CfVam7-7F/GFP-R were used for PCR identification of positive clones. The successfully fused plasmids were then transformed into the *Escherichia coli* JM109 competent cells. PCR was used for the identification and sequencing of *E. coli*-positive clones. The complementary carriers with correct sequences were transformed into the protoplasts of Δ*Cfvam7*. The transformants that could grow on a BLE-containing culture medium were screened by fluorescent microscopy and further confirmed by PCR (Yao et al., 2019).

For domain deletion strains, the genomic DNA of the wild-type CFLH16 strain was used as the template. The primers CfVam7-9F/CfVam7-PXR1 and CfVam7-PXF2/CfVam7-10R were used for the amplification of DNA fragments when constructing the PX domain deletion vector. Primers CfVam7-9F/CfVam7-SNR were used for the amplification of DNA fragments of SNARE domain deletion vector. The sequences of the primers are shown in Supplementary Table 1. After purification, the PCR product was co-transformed into the yeast competent cell XK-125 along with linearized pYF11 vector to finally acquire the complementary carriers with domain deletion, namely, pYF11:*CfVAM7*^Δ*PX*^ and pYF11:*CfVAM7*^Δ*SNARE*^. *E. coli* plasmid vectors with correct sequencing results were transformed into the protoplast of mutant strain Δ*Cfvam7*. The transformants were subjected to BLE resistance screening and fluorescent observation, and the complementary strains with domain deletion were finally acquired.

### Phenotype Analysis

For growth rate, the wild-type strain, mutant strain Δ*Cfvam7*, domain deletion strains, and complementary strain were cultured on the PDA and minimal medium (MM) medium at 28°C for 3 days, and then the diameters of colonies were measured for statistical analysis. Each strain was prepared in triplicate, and the experiments were repeated three times.

For sporulation and appressorium formation, the wild-type strain, mutant strain Δ*Cfvam7*, and complementary strain were cultured in potato dextrose broth (PDB) at 28°C, 160 rpm for 3 days. The sporulation quantity was measured using the hemocytometer under a microscope, which was used for data analysis. For appressorium formation assays, the conidium of the strains was collected, and the concentration was adjusted to 1 × 10^5^/ml. Then 10 μl of the conidium suspension was added to hydrophobic slides and cultured in wet at 28°C for 24 h. The formation of appressorium was subjected to microscopic observation for statistical analysis.

For stress sensitivity, the abovementioned strains were cultured with PDA medium containing the cell wall stressor 0.01% sodium dodecyl sulfate (SDS; Macklin, Shanghai, China; 151-21-3), 200 μg/ml of calcofluor white (CFW; Shyuanye, Shanghai, China; 4404-43-7), 0.05% Congo red (CR; Hushi, Shanghai, China; 573-58-0), and ER stress inducer [5 mM DTT; Solarbio, Beijing, China; 3483-12-3; 0.5 μg/ml of tunicamycin (TM); Solarbio; 11089-65-9] for 3 days. The diameters of colonies were measured, and the inhibition rate was estimated.

### Pathogenicity Assays

The conidium suspensions of the above strains with a concentration of 1 × 10^5^/ml were inoculated on the margins of injured and non-injured isolated leaves of *Ca. oleifera* and cultured in an incubator with moisture for 12 h to observe the disease.

### Vacuole Fusion Tests

The hyphae of the abovementioned strains were placed in a PDB culture medium and cultured at 28°C, 160 rpm, for 2 days. Next, neutral red (Solarbio; G1310) staining was performed on the hyphae of the three strains for 5–10 min, followed by processing using 0.5 M of NaCl solution for 15 min. Then the hyphae were further treated using sterilized water for 2–4 h. Finally, the results of each process were observed by fluorescence microscopy (ZEISS, Oberkochen, Germany; Axio Observer.A1).

### Statistical Analysis

All statistical data were expressed as mean ± standard deviation (SD) and analyzed by a one-way ANOVA and Duncan’s new multiple-range test.

## Results

### Identification of CfVam7 in *Colletotrichum fructicola*

The qRT-PCR results showed that the expression of A11731 gene in the mutant strain Δ*Cfhac1* was significantly reduced (Supplementary Figure 1), which was in agreement with the transcriptome sequencing results. This further indicated that the expression of A11731 in *C. fructicola* was regulated by transcription factor CfHac1.

**FIGURE 1:**
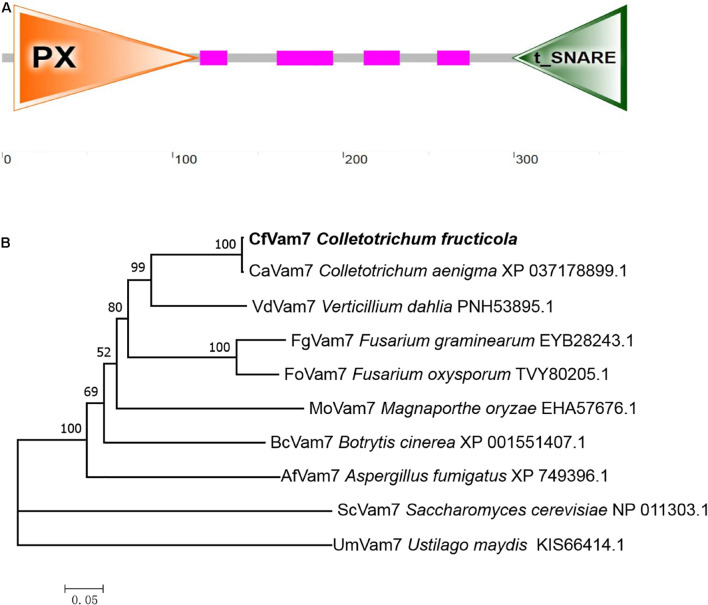
Domain prediction and phylogenetic analysis of CfVam7. **(A)** The domain prediction of CfVam7. The orange triangle (7–115 amino acids) indicates the PHOX (PX) domain, and the green triangle (299–366 amino acids) refers to the t_SNARE domain. **(B)** The phylogenetic tree was constructed using MEGA 7.0 and the neighbor-joining method with 1,000 bootstrap replicates.

The full length of A11731 gene was 1,101 bp, which encoded a protein with 366 amino acids. Domain prediction analysis revealed a PHOX (PX) domain (7–115 residues) at A11731 gene N-terminal and a t-SNARE domain (299–366 residues) at A11731 gene C-terminal (Figure 1A). The amino acid sequences encoded by A11731 gene were orthologous to the ScVam7 protein of *S. cerevisiae*; thus, the gene was named *CfVAM7*. To investigate the conservation of the amino acid sequences of CfVam7, the orthologous proteins of other fungi were obtained from the NCBI database for comparative analysis and phylogenetic tree construction. These findings showed that the CfVam7 protein of *C. fructicola* had high homology with the CaVam7 protein of *Colletotrichum aenigma* (identity: 99.73%) and low homology with the ScVam7 protein of *S. cerevisiae* (identity: 23.38%) (Figure 1B).

*CfVAM7* gene was knocked out by homologous recombination, and the strategy is shown in Supplementary Figure 2A. PCR verification of transformants showed that using the inner primer CfVam7-7F/CfVam7-8R could not amplify the target strip of the DNA in the mutant strain Δ*Cfvam7* but could amplify the target DAN strip in the wild-type positive control strain. In contrast, using the outer primer CfVam7-5F/H855R could amplify the target strip of the DNA in the mutant strain Δ*Cfvam7*, but not in the wild-type positive control strain. These findings indicated that *CfVAM7* gene was successfully knocked out. The pYF11:*CfVAM7* fusion vector with the promoter region was further constructed, and the vectors with correct sequencing results were transformed to the mutant strain Δ*Cfvam7* to acquire the complementary strain Δ*Cfvam7*/*CfVAM7* (Supplementary Figure 2B).

### CfVam7 Regulates Vegetative Growth, Conidiation, and Appressorium Formation

**FIGURE 2:**
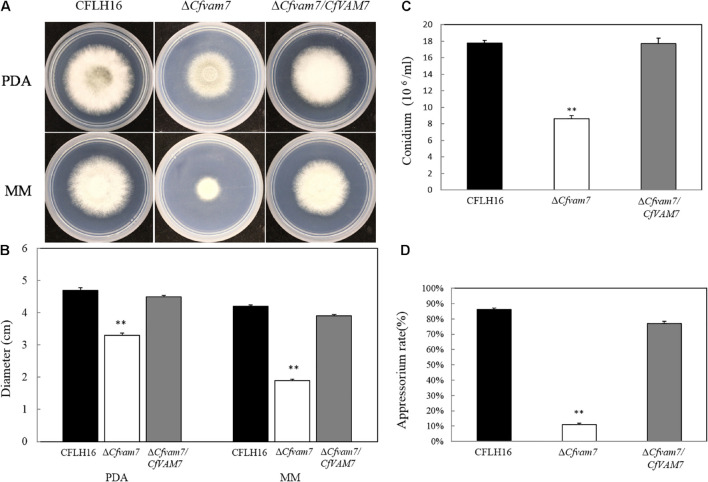
CfVam7 is involved in vegetative growth, sporulation, and appressorium formation. **(A)** Growth rate of strains on potato dextrose agar (PDA) and MM media. **(B)** Statistical analysis of the colony diameter variations. **(C)** Conidium formation rate. **(D)** Appressorium rate. Error bars are standard deviation, and asterisks represent significance at *p* < 0.01 (^∗∗^).

To explore the role of *CfVAM7* gene in the vegetative growth of *C. fructicola*, the growth of the mutant strain Δ*Cfvam7* on two different culture mediums, PDA and MM, was investigated. Our results showed significantly lower diameters of Δ*Cfvam7* than did the wild-type CFLH16 and complementary strain Δ*Cfvam7*/*CfVAM7* on both culture mediums (*p* < 0.01), indicating that *CfVAM7* gene participated in the regulation of vegetative growth of *C. fructicola* (Figures 2A,B).

Next, the conidiation of the mutant strain Δ*Cfvam7* was analyzed, revealing a significant reduction of about 50% of the sporulation ability of wild-type and complementary strains (Figure 2C). Further investigation showed that the rate of appressorium formation of mutant strain Δ*Cfvam7* was about only 10%, which was significantly lower than that of the wild-type and complementary strains (*p* < 0.01) (Figure 2D). These findings demonstrated that *CfVAM7* gene was important for vegetative growth, conidiation, and appressorium formation of *C. fructicola*.

### CfVam7 Regulates Pathogenicity of *Colletotrichum fructicola*

**FIGURE 3:**
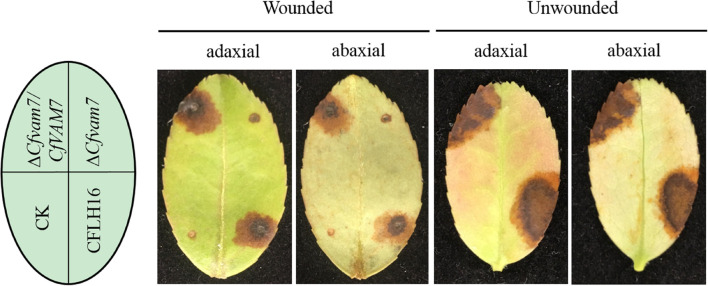
CfVam7 is essential for pathogenicity. Unwounded and wounded tea-oil tree leaves were inoculated with conidium suspensions of wild type (WT), Δ*Cfvam7*, and Δ*Cfvam7*/*CfVAM7*. Up, up of the leaf; Down, down of the leaf; CK: compared with the control, the agar plug or ddH_2_O was inoculated onto it.

The appressorium formed by conidium is an important structure for host infection by plant pathogenic fungi. Our results showed that *CfVAM7* gene participated in the regulation of appressorium formation in *C. fructicola*. However, it was still unclear whether the pathogenicity of mutant strain Δ*Cfvam7* was influenced. Our results showed that the lesion area in the mutant strain Δ*Cfvam7* was significantly lower than in the wild-type and complementary strains (*p* < 0.01). Furthermore, when seeded to the leaves without wound for 5 days, the pathogenicity of the mutant strain was completely lost compared with that in the wild-type and complementary strains (Figure 3). These findings demonstrated that CfVam7 is essential for *C. fructicola* pathogenicity.

### *CfVAM7* Gene Participated in the Responses of *Colletotrichum fructicola* to Cell Wall Stress

**FIGURE 4:**
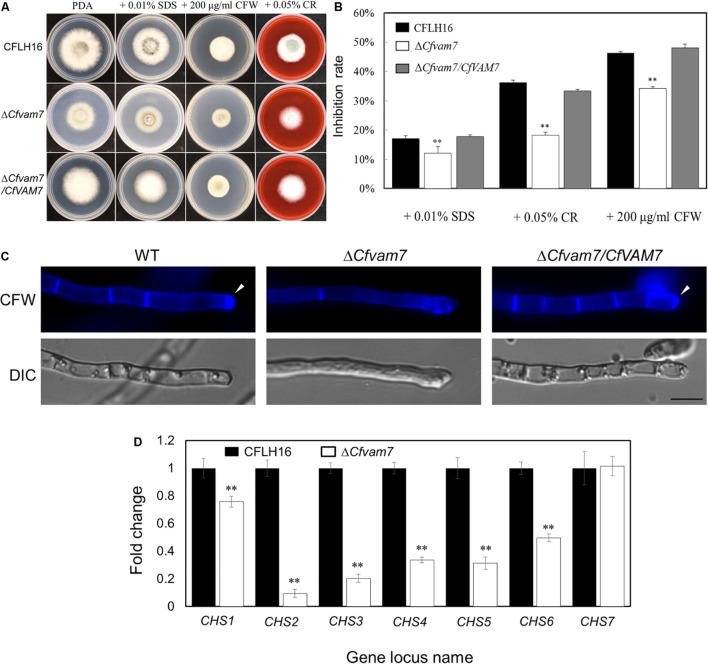
CfVam7 plays roles in cell wall integrity. **(A)** The wild type (WT), Δ*Cfvam7*, and Δ*Cfvam7*/*CfVAM7* were incubated on potato dextrose agar (PDA) plates with various cell wall stresses of calcofluor white (CFW), Congo red (CR), and sodium dodecyl sulfate (SDS) at 28°C. **(B)** Statistical analysis of inhibition rates of the strains to cell wall stresses, and asterisks indicate significant differences (*p* < 0.01). **(C)** The mycelia of the strains were stained with 10 mg/ml of CFW for 5 min without light before being photographed; arrows indicate the stained hyphal tips. The experiment was repeated three times with triplicates, which showed the same results. DIC, differential interference contrast image. **(D)** Reduced expression was found in six out of seven genes that encode chitin synthases in the Δ*Cfvam7* mutants of *Colletotrichum fructicola*. RNA was extracted from mycelia that were grown for 3 days in liquid potato dextrose broth (PDB). Error bars represent the standard deviation, and “**” represent significant difference among stains tested. All of the reductions are significant (*p* = 0.01 or *p* = 0.05) according to Duncan’s multiple-range test.

The growth and development of *C. fructicola* were influenced by various cell wall stressors in natural conditions. In this study, we investigated the sensitivity of the mutant strain Δ*Cfvam7* to cell wall stressors (including SDS, CFW, and CR). The results showed that the inhibition rate of the mutant strain Δ*Cfvam7* was significantly reduced as compared with that of the wild-type and complementary strains (*p* < 0.01) (Figures 4A,B). Our results suggested that *CfVAM7* gene participated in the responses of *C. fructicola* to cell wall stress. CFW staining was used to assess the distribution of chitinous substances at the tip of Δ*Cfvam7* hyphae, revealing that the chitins were mainly aggregated at the hyphal tip in the wild-type and complementary strains. Nonetheless, in the mutant mycelium, almost no chitins aggregated at the hyphal tip and was with longer distance between two septa, which indicated that the knockout of *CfVAM7* gene influenced the distribution of mycelial chitin (Figure 4C). Further analysis by qRT-PCR on the expression of seven chitin synthases genes, namely, *CHS1*, *CHS2*, *CHS3*, *CHS4*, *CHS5*, *CHS6*, and *CHS7*, in the mutant strain Δ*Cfvam7* showed that the expressions of six chitin synthases were significantly downregulated (*p* < 0.01) (Figure 4D), suggesting that *CfVAM7* gene participated in the regulation of chitin synthase gene expression in *C. fructicola*.

### *CfVAM7* Gene Participated in the Responses of *Colletotrichum fructicola* to Endoplasmic Reticulum Stress

**FIGURE 5:**
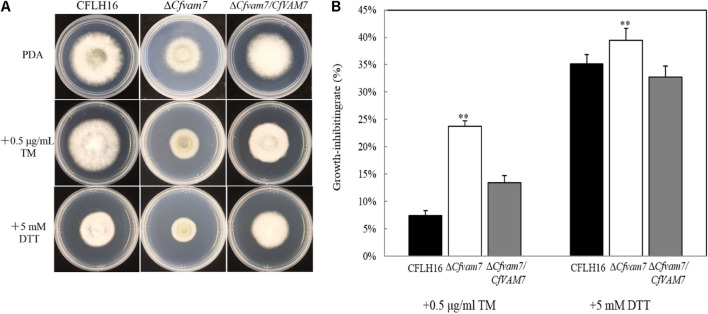
CfVam7 participates in the responses to endoplasmic reticulum (ER) stresses. **(A)** The wild type (WT), Δ*Cfvam7*, andΔ*Cfvam7*/*CfVAM7* were incubated on potato dextrose agar (PDA) plates with 0.5 μg/ml of tunicamycin (TM) and 5.0 mM of dithiothreitol (DTT) at 28°C. **(B)** Statistical analysis of inhibition rates of the strains to TM and DTT stress. Error bars represent SD of three replicates, and “**” indicate significant difference (*p* < 0.01).

Dithiothreitol and TM are two ER stress factors altering protein folding through different mechanisms. DTT blocks disulfide bond formation, and TM blocks *N*-glycosidic protein–carbohydrate linkage formation (Qu et al., 2020). We investigated the growth of the mutant strain Δ*Cfvam7* in a culture medium containing ER stressors (DTT and TM). Our results showed that compared with the growth rate of the wild-type and complementary strains, the growth rate of mutant strain Δ*Cfvam7* was significantly slower in the PDA culture medium with DTT and TM stressors. The inhibition rate analysis showed that the resistance of mutant strain to DTT and TM was significantly reduced (*p* < 0.01) (Figures 5A,B). These findings suggested that the responses of mutant strain Δ*Cfvam7* to ER stress changed and *CfVAM7* gene participated in regulating the responses of *C. fructicola* to ER stress.

### *CfVAM7* Gene Participated in the Vacuole Fusion of *Colletotrichum fructicola*

**FIGURE 6:**
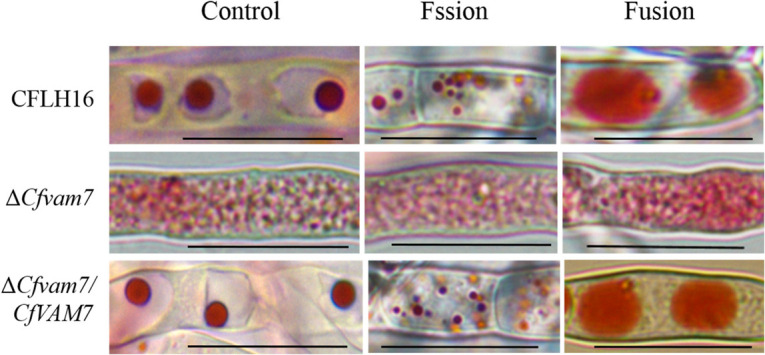
CfVam7 regulates vacuole fusion. Vacuoles were labeled with neutral red and incubated for 15 min in potato dextrose broth (PDB) medium plus 0.5 M of NaCl for fission experiments. For fusion experiments, hyphae were further incubated for 2–4 h in water. Scale bar = 20 μm.

Vesicular trafficking is ubiquitously present in the vital functions of organisms, and SNARE protein plays an important role in the fusion between vesicles and the membrane of target cells (Marino and Heidi, 2001). To investigate whether *CfVAM7* gene deletion could influence the fusion of vacuole membrane, neutral red staining was used to evaluate the processes of vacuole fusion. Relatively large elliptical vacuoles, arranged regularly, with a diameter of about 3–5 μm were found in the wild-type and complementary strains. In contrast, the hyphal cell of the mutant strain Δ*Cfvam7* was filled with small vesicles with a diameter of <0.5 μm, and there were no large vacuoles. To further investigate the role of CfVam7 in regulating the formation of vacuoles, 0.5 M of NaCl solution and sterilized water were used to assess the fission and fusion of vacuoles. The results revealed that after treatment with NaCl solution, more vacuoles with smaller sizes were found in the wild-type and complementary strains, with a diameter of about 1–2 μm. The number and sizes of vacuoles in the mutant strain Δ*Cfvam7* did not significantly change. After further short-term treatment in sterilized water, the vacuoles of the wild-type and complementary strains fused into large vacuoles with a diameter of >8 μm, but the vesicles of the mutant strain Δ*Cfvam7* did not fuse (Figure 6). These findings demonstrated that CfVam7 participated in regulating the fusion of vacuoles in *C. fructicola*.

### The Phox Homolog and SNARE Domains Were Important for the Function of CfVam7

**FIGURE 7:**
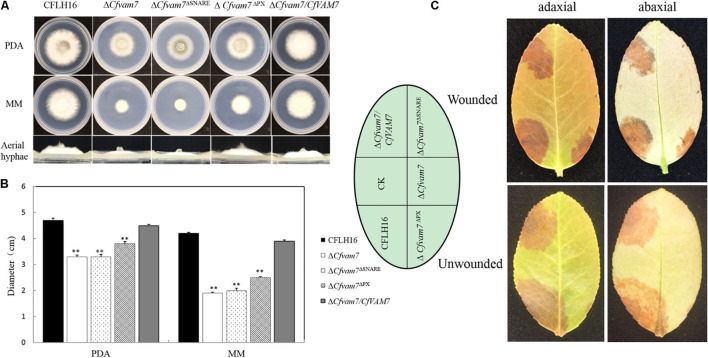
The Phox homology (PX) and SNARE domain is important for the function of CfVam7. **(A)** Growth of wild type (WT), Δ*Cfvam7*, Δ*Cfvam7*^Δ*SNARE*^, Δ*Cfvam7*^Δ*PX*^, and Δ*Cfvam7*/*CfVAM7* on potato dextrose agar (PDA) and MM plates. **(B)** Statistical analysis of colony diameters of the strains. Error bars represent SD of three replicates, and “**” indicate significant difference (*p* < 0.01). **(C)** Diseased symptoms of wounded and unwounded tea-oil tree leaves inoculated with related mycelial plugs.

CfVam7 protein consists of a SNARE and a PX domain. To explore the functions of the domains, two vectors with the deletion of SNARE or PX domain were constructed and transformed into the mutant strain Δ*Cfvam7* according to the protoplast transformation method. BLE-resistant transformants were acquired, and the strains Δ*Cfvam7*^Δ*SNARE*^ and Δ*Cfvam7*^Δ*PX*^ with green fluorescence were screened. These strains with the absence of these two domains were investigated, and the findings showed that compared with the wild-type and complementary strains, the strain with SNARE domain deletion could only produce a low amount of aerial hypha, while the growth rate was significantly reduced, which was similar with the Δ*Cfvam7* mutant (*p* < 0.01) (Figures 7A,B). On the contrary, the colonies of the PX domain deletion strain showed moderate growth rates between wild type and Δ*Cfvam7* mutant, while the aerial hypha was not significantly influenced (Figures 7A,B). The pathogenicity test showed that the SNARE domain deletion strain could not induce disease spots on the leaves of *Ca. oleifera* with or without wound, similar to the mutant strain Δ*Cfvam7* (Figure 7C). The PX domain deletion strain could induce disease spots on a leaf with or without a wound. However, the sizes of the disease spots were significantly lower compared with those of wild-type and complementary strains (*p* < 0.01) (Figure 7C). These findings demonstrated that the both SNARE and PX domains of *CfVAM7* gene participated in the regulation of growth and pathogenicity of *C. fructicola*.

### The Phox Homolog and SNARE Domain Contributed to the Vacuolar Membrane Localization of CfVam7

**FIGURE 8:**
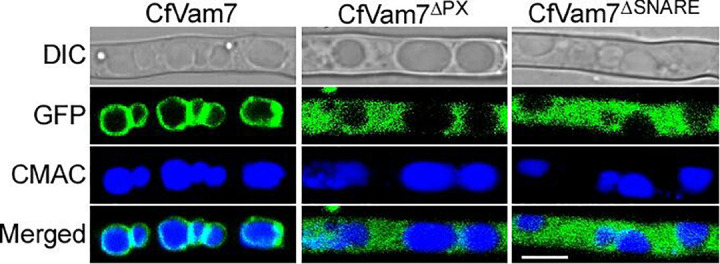
The Phox homology (PX) and SNARE domain contributes to the vacuolar membrane localization of CfVam7. Hyphae of GFP-CfVam7 strain were incubated on liquid medium for 24 h. 7-Amino-4-chloromethylcoumarin (CMAC) staining of vacuole was performed at the 37°C for 30 min. Photographs were examined under differential interference contrast (DIC) or epifluorescence microscopy. The merged panels showed the CfVam7 localizes to vacuolar membrane in Δ*Cfvam7*/*CfVAM7*, and the subcellular localization of GFP-CfVam7^Δ*SNARE*^ and GFP-CfVam7^Δ*PX*^ proteins changed. Bar = 10 μm.

The subcellular localization of CfVam7 protein was investigated in the complete complementary strain Δ*Cfvam7*/*CfVAM7*, as well as the complementary strains Δ*Cfvam7*^Δ*SNARE*^ and Δ*Cfvam7*^Δ*PX*^ with SNARE and PX domain deletion, respectively. The results showed that in the complete complementary strain Δ*Cfvam7*/*CfVAM7*, the green fluorescence of GFP-CfVam7 was co-localized with the vacuole membrane of mature hypha as ring-shaped structures, indicating that CfVam7 protein was localized at the vacuole membrane in mature hyphae. In the domain absent complementary strains, the subcellular localization of GFP-CfVam7^Δ*SNARE*^ and GFP-CfVam7^Δ*PX*^ proteins changed (Figure 8). Specifically, the green fluorescence was evenly distributed in the cytoplasm of mature hyphae, indicating that the CfVam7^Δ*SNARE*^ and CfVam7^Δ*PX*^ proteins were not correctly localized to the vacuole membrane of hyphae in domain deletion strains to exert the functions (Figure 8). These findings demonstrated that the SNARE and PX domains participated in the subcellular localization of CfVam7 protein, consequently influencing its biological functions.

## Discussion

*Camellia oleifera* is a ligneous plant native to China, which produces edible oil materials. Anthracnose is the major disease of *Ca. oleifera*, which causes substantial financial loss (Zhang et al., 2021). While *C. fructicola* has been identified as the prominent pathogen causing *Ca. oleifera* anthracnose, the underlying pathogenic mechanisms remain unclear.

Vam7 is a t-SNARE protein, which is involved in regulating the growth, development, and pathogenicity of plant pathogens. Dou et al. (2011) investigated the biological functions of the SNARE protein MoVam7 in *Magnaporthe oryzae*. They found that MoVam7 not only participated in maintaining the shapes and functions of vacuoles but also contributed to the regulation of cell wall integrity, endocytosis, reactive oxygen species accumulation, production of conidium, and pathogenicity. Zhang and colleagues reported that FgVam7 participated in regulating vesicle trafficking, consequently influencing the growth, development, syngenesis, vomitoxin (DON) production, and pathogenicity of *Fusarium graminearum* (Zhang et al., 2016). Li et al. (2019b) investigated the FolVam7 of *Fusarium oxysporum* and found that this protein could influence the cellular material transportation, thereby regulating the growth, development, and pathogenicity of the fungus. In this study, a SNARE protein, CfVam7, was identified in *C. fructicola*, which was the prominent pathogen of *Ca. oleifera* anthracnose. Our results showed that this protein participated in hyphal growth, sporogenesis, appressorium formation, responses to stresses, vacuole fusion, and pathogenicity, providing evidence for further investigation of the mechanisms underlying the pathogeneses of *C. fructicola*. CfVam7 might be a potential target for developing new fungicides.

Endoplasmic reticulum is the cellular organelle for the synthesis of a series of biomacromolecules such as proteins, lipids (e.g., triglyceride), and saccharides, rather than nucleic acids. Overaccumulation of misfolded or unfolded proteins, or dysregulation of sterols or lipids, could trigger the ER stress and influence the expression of specific genes. Such responses are known as unfolded protein responses (UPRs) (Tang et al., 2015; Goh et al., 2017). UPR is essential for the pathogenesis of pathogens, and bZIP transcription factor Hac1 participates in the processes of UPR (Tang et al., 2015). Our previous studies have demonstrated that the bZIP transcription factor CfHac1 could regulate DTT-induced ER stress responses and pathogenicity of *C. fructicola* (Yao et al., 2019; Li and Li, 2020). Further transcriptome analysis in *CfHAC1* gene knockout *C. fructicola* strain under DTT stress showed that the downregulated genes were mainly concentrated at the pathways of ER protein processing, biosynthesis of N-glycan, synthesis of steroids, and protein secretion associated with ER functions. In particular, there was no expression of CfVam7 protein encoded by A11731 gene in the mutant strain Δ*Cfhac1*, which suggested that the expression of *CfVAM7* gene was regulated by the transcription factor CfHac1 (Li and Li, 2020). For the first time, this study revealed that CfVam7 protein regulated the responses of *C. fructicola* to ER stress, which was consistent with previous findings. The other studies also showed that transcription factor CfHac1 responded to ER stress, which could be associated with the transcriptional regulation of *CfVAM7* gene expression by CfHac1 in ER stress response process. Still, the exact mechanisms underlying the regulation of ER stress responses need to be further investigated.

In addition, stress induction experiments using cell membrane inhibitor SDS and cell wall inhibitors CFW and CR showed that the growth inhibition rate of the mutant strain Δ*Cfvam7* was significantly lower than that of the wild-type complementary strains. Chitin is a major component of fungal cell wall and is synthesized by chitin synthases; the reduced chitin synthase gene expression may cause less sensitivity to the cell wall stresses. However, more studies are needed to further investigate whether these findings are associated with the absence of normal vacuoles in the mutant strain.

**FIGURE 9:**
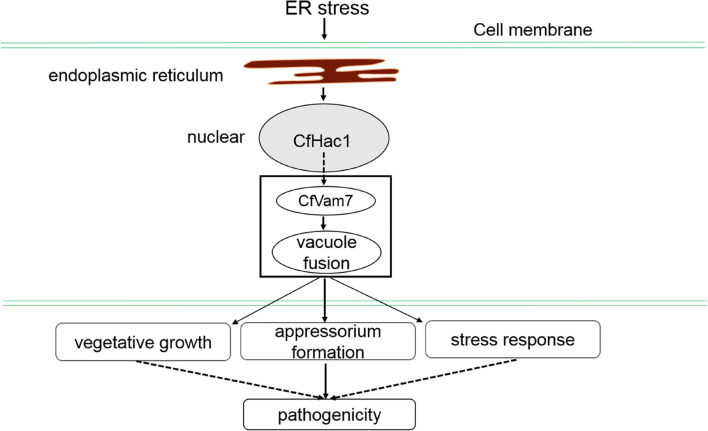
The working model of Cfvam7 in *Colletotrichum fruiticola*.

The PX domain is a phospholipid-binding domain that consists of 120 amino acids. Previous studies have demonstrated the structures of various proteins consisting of a specific PX domain, through which the protein could bind to membrane phosphatidylinositol (PI) and anchor the protein to the cell membrane, exerting the sorting, transporting, and signal transduction functions (Pan and Xu, 2002). Sato et al. (2001) reported that the PX domain of yeast Vam7 protein could bind to PI_3_P in endosomes. In addition, Vam7 protein was also identified in vacuoles of yeasts. Further investigations showed that Vam7 consisted of an α-helix of SNARE, which could recognize the t-SNARE of endogenous Vam3 protein on vacuole membrane, allowing Vam7 protein to transport from endosome to vacuoles. The findings of this study demonstrated that the absence of either PX or SNARE domain could lead to mislocalization of CfVam7 protein, which could be the major cause of the substantially reduced growth, development, and pathogenicity of the domain absent strains. In addition, this study also demonstrated that the pathogenicity of SNARE domain deletion strain was completely abrogated, while the PX domain deletion strain still preserved weak pathogenicity, and the absence of PX domain did not influence the growth of aerial hypha of the strain. For the working model of Cfvam7 in *C. fruiticola*, please see Figure 9. More studies are needed to further investigate the exact mechanisms.

## Data Availability Statement

The original contributions presented in the study are included in the article/Supplementary Material, further inquiries can be directed to the corresponding author.

## Author Contributions

HL and SL conceived and designed the study and wrote the manuscript. SL, BL, and SZ performed the experiments. All authors contributed to the article and approved the submitted version.

## Conflict of Interest

The authors declare that the research was conducted in the absence of any commercial or financial relationships that could be construed as a potential conflict of interest.

## Publisher’s Note

All claims expressed in this article are solely those of the authors and do not necessarily represent those of their affiliated organizations, or those of the publisher, the editors and the reviewers. Any product that may be evaluated in this article, or claim that may be made by its manufacturer, is not guaranteed or endorsed by the publisher.

## Footnotes

1
https://www.ncbi.nlm.nih.gov/

2
http://smart.embl-heidelberg.de/

## Supplementary Material

Supplementary Figure 1 qRT-PCR analysis of expression levels of A11731 gene in Δ*Cfhac1*.

Supplementary Figure 2 Generation of the *CfVAM7* gene deletion mutant in *C. fructicola*. **(A)** Schematic diagram of the deletion strategy for the *CfVAM7* gene. **(B)** Primer1: *CfVAM7*-5F/H855R; primer2: *CfVAM7*-7F/*CfVAM7*-8R; M: DL2000 marker; -: H_2_O negative control; +: WT positive control; Δ: Mutant; C: Complemented strain.

Supplementary Table 1 Primers used in this study.
