# Supplementary figures and images for: The SNARE Protein CfVam7 Is Required for Growth, Endoplasmic Reticulum Stress Response, and Pathogenicity of Colletotrichum fructicola
Source: Front Microbiol. 2021 Oct 14;12:736066. doi: 10.3389/fmicb.2021.736066 (PMC8551764; doi:10.3389/fmicb.2021.736066)

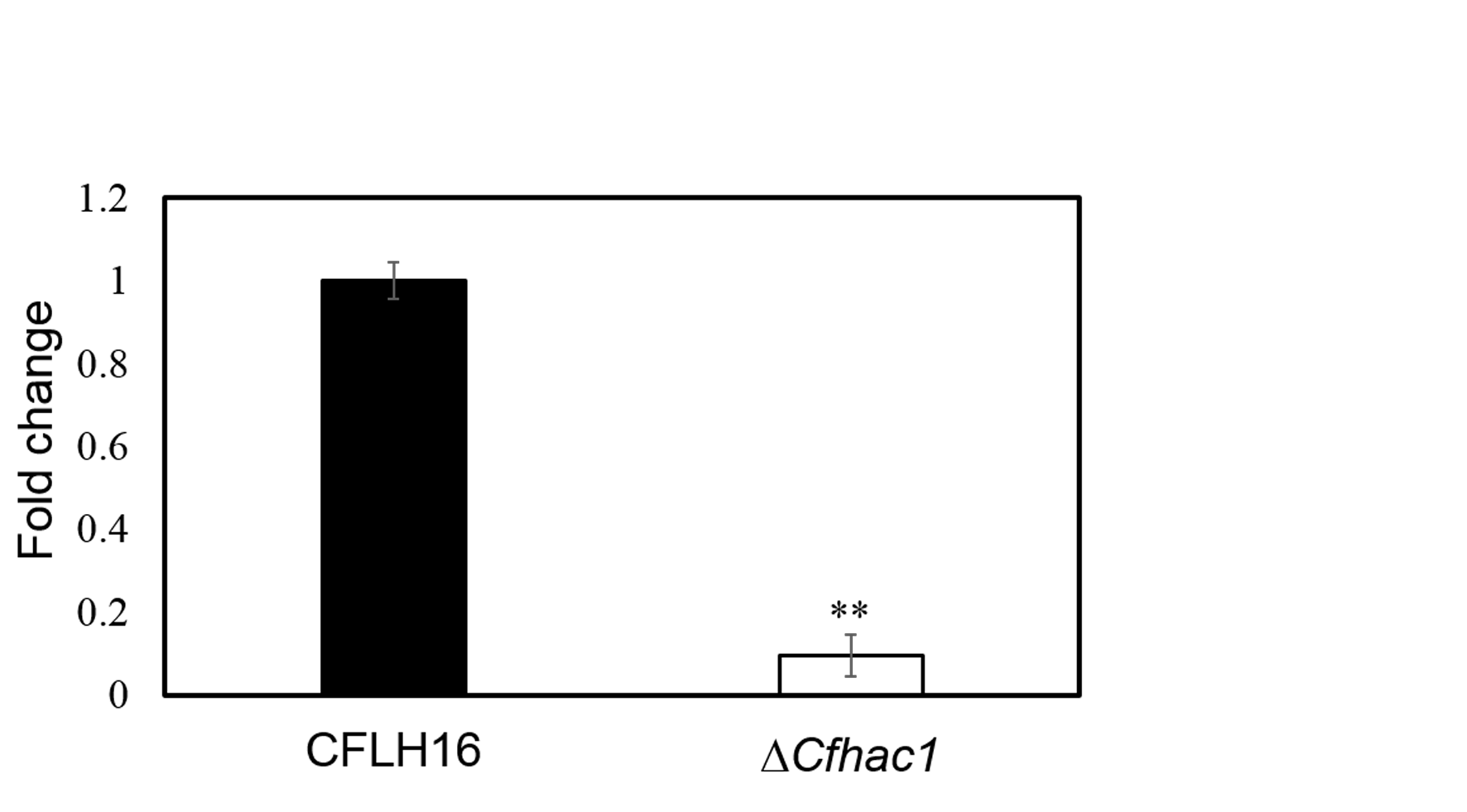

Supplement: Supplementary Figure 1 — qRT-PCR analysis of expression levels of A11731 gene in ΔCfhac1. [file Image_1.TIF]

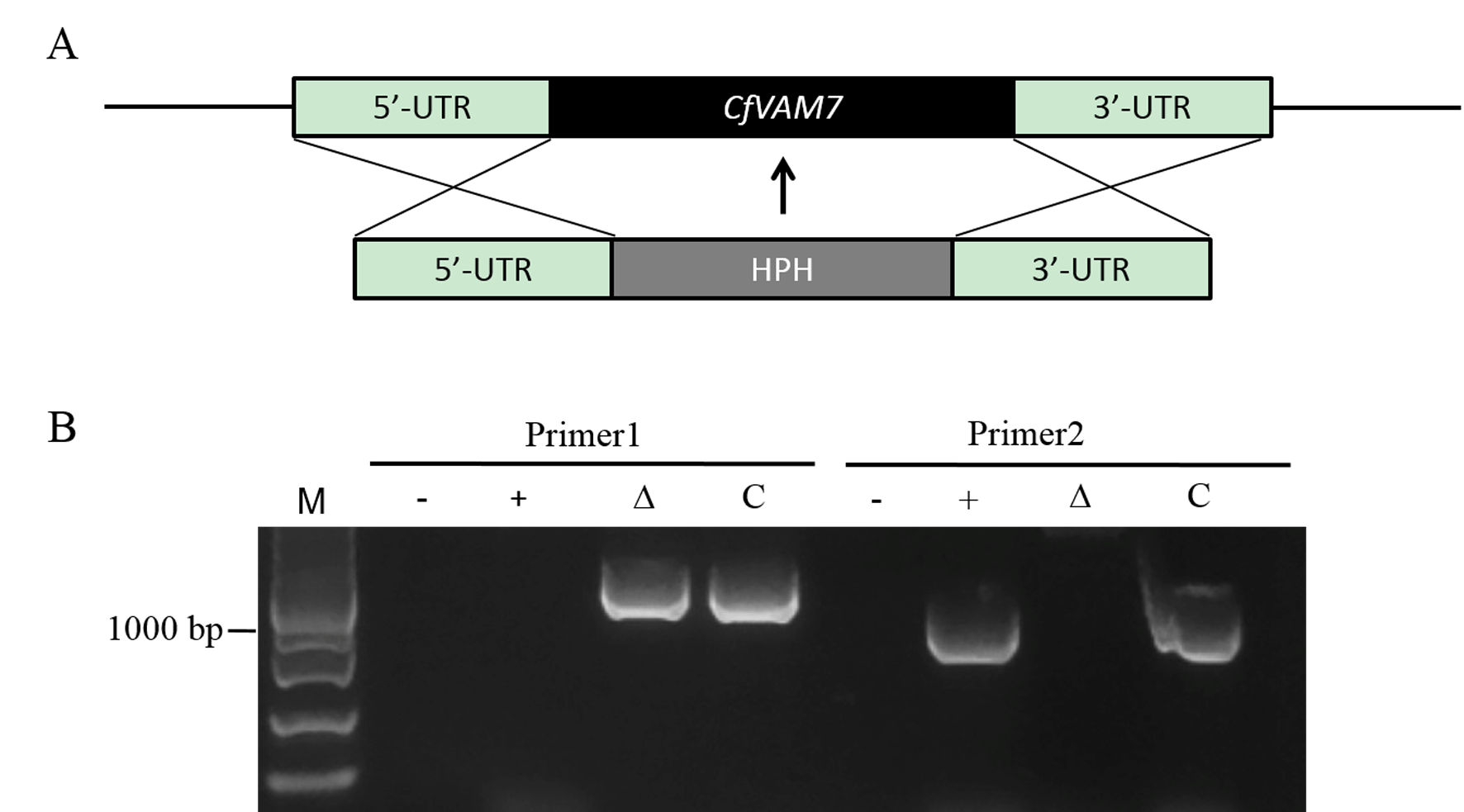

Supplement: Supplementary Figure 2 — Generation of the CfVAM7 gene deletion mutant in C. fructicola. (A) Schematic diagram of the deletion strategy for the CfVAM7 gene. (B) Primer1: CfVAM7-5F/H855R; primer2: CfVAM7-7F/CfVAM7-8R; M: DL2000 marker; -: H2O negative control; +: WT positive control; Δ: Mutant; C: Complemented strain. [file Image_2.TIF]
