# Supplementary material for: The SNARE Protein CfVam7 Is Required for Growth, Endoplasmic Reticulum Stress Response, and Pathogenicity of Colletotrichum fructicola
Source: Front Microbiol. 2021 Oct 14;12:736066. doi: 10.3389/fmicb.2021.736066 (PMC8551764; doi:10.3389/fmicb.2021.736066)
Supplement: Supplementary Table 1 — Primers used in this study. [file Data_Sheet_1.PDF]

Table 1 Primers used in this study

Primer	Sequence（5'→3'）	Purpose
CfVam7-1F	TACTAGTCGTCGGAGAGGTG	amplify *CfVAM7* 5' flank sequence
CfVam7-2R	TTGACCTCCACTAGCTCCAGCCAAGCC TGTGACGGCGGTTGTTGTGA	amplify *CfVAM7* 5' flank sequence
CfVam7-3F	CAAAGGAATAGAGTAGATGCCGACCG GGCGCATCGGCAAGTTCTGA	amplify *CfVAM7* 3' flank sequence
CfVam7-4R	CCTGGTTGGATTTACTTCAA	amplify *CfVAM7* 3' flank sequence
CfVam7-5F	AGACGGCTCAGACGATTCGT	validation of *CfVAM7* gene deletion
H855R	GCTGATCTGACCAGTTGC	validation of *CfVAM7* gene deletion
CfVam7-7F	AGCCCTACACCCTCTACAACA	amplify *CfVAM7* gene sequence
CfVam7-8R	AGCCCTACACCCTCTACAACA	amplify *CfVAM7* gene sequence
CfVam7-9F	ACTCACTATAGGGCGAATTGGGTACTCAAAT TGGTTCAGGAGACCTTTTAGGCGGGTGG	amplify complemented sequence
CfVam7-10R	CACCACCCCGGTGAACAGCTCCTCGCCCTTG CTCACGAACTTGCCGATGCGCCGGTTC	amplify complemented sequence
Hyg-F	GGCTTGGCTGGAGCTAGTGGAGGTCAA	amplify *HPH* sequence
Hyg-R	CGGTCGGCATCTACTCTATTCCTTTG	amplify *HPH* sequence
CfVam7-PXR1	CTCGGCCGGGGGAGCCATTG	amplify ΔPX complemented sequence
CfVam7-PXF2	CAATGGCTCCCCCGGCCGAGAGCACCGGCGG CGGCGGCAGCACGA	amplify ΔPX complemented sequence
CfVam7-SNR	CACCACCCCGGTGAACAGCTCCTCGCCCTTG CTCACCCCGTCGTTGTCCAGCT	amplify ΔSNARE sequence
GFP-R	GACACGCTGAACTTGTGGCCGTT	amplify complemented sequence
